# Spheno-Occipital Synchondrosis Fusion Correlates with Cervical Vertebrae Maturation

**DOI:** 10.1371/journal.pone.0161104

**Published:** 2016-08-11

**Authors:** María José Fernández-Pérez, José Antonio Alarcón, James A. McNamara, Miguel Velasco-Torres, Erika Benavides, Pablo Galindo-Moreno, Andrés Catena

**Affiliations:** 1 Department of Orthodontics, School of Dentistry, University of Granada, Granada, Spain; 2 Department of Orthodontics and Pediatric Dentistry, School of Dentistry, The University of Michigan, Ann Arbor, Michigan, United States of America; 3 Department of Oral Surgery and Implant Dentistry, School of Dentistry, University of Granada, Granada, Spain; 4 Department of Periodontics and Oral Medicine, School of Dentistry, University of Michigan, Ann Arbor, Michigan, United States of America; 5 Mind, Brain, and Behavior Research Center, University of Granada, Granada, Spain; University of Kentucky, UNITED STATES

## Abstract

The aim of this study was to determine the relationship between the closure stage of the spheno-occipital synchondrosis and the maturational stage of the cervical vertebrae (CVM) in growing and young adult subjects using cone beam computed tomography (CBCT). CBCT images with an extended field of view obtained from 315 participants (148 females and 167 males; mean age 15.6 ±7.3 years; range 6 to 23 years) were analyzed. The fusion status of the synchondrosis was determined using a five-stage scoring system; the vertebral maturational status was evaluated using a six-stage stratification (CVM method). Ordinal regression was used to study the ability of the synchondrosis stage to predict the vertebral maturation stage. Vertebrae and synchondrosis had a strong significant correlation (r = 0.89) that essential was similar for females (r = 0.88) and males (r = 0.89). CVM stage could be accurately predicted from synchondrosis stage by ordinal regression models. Prediction equations of the vertebral stage using synchondrosis stage, sex and biological age as predictors were developed. Thus this investigation demonstrated that the stage of spheno-occipital synchondrosis, as determined in CBCT images, is a reasonable indicator of growth maturation.

## Introduction

To estimate accurately the skeletal maturity of both growing subjects and young adults has been a challenging task in different areas of dentistry, most commonly in orthodontics (e.g. for planning the appropriate time for rapid maxillary expansion or functional treatments) and surgery (e.g. for planning treatment timing for orthognathic surgery or endosseous implants). In the past, several methods have been used to determine skeletal age. Hand-wrist radiographs have been proposed to estimate a patient’s bone maturation stage with relatively high accuracy [1–3]. However, this methodology presents several drawbacks, including the additional amount of radiation required.

To address these limitations, an alternate method has been developed to analyse patient age through the maturation of the cervical vertebra, termed the CVM method. The same lateral cephalogram that is used routinely for orthodontic diagnosis and treatment planning contains additional information that can be used diagnostically. The size and shape of the 2^nd^, 3^rd^ and 4^th^ cervical vertebrae can be used to estimate the stage of maturational development of the craniofacial region, thus eliminating the need for an additional radiograph [4, 5]. More recently, middle phalanx maturation (MPM) of the third finger has been proposed as a valid indicator of the pubertal growth spurt in individual subjects; both the MPM and CVM methods have demonstrated an overall satisfactory diagnostic agreement, although with a slight disagreement at stage 5, in which the third middle phalanx appears to mature earlier than the cervical vertebrae [6].

The maturational age of a subject can also be estimated by analyzing the closure of the spheno-occipital synchondrosis, which is located in the posterior part of the cranial base (clivus), anterior to the foramen magnum and inferior to sella turcica. This synchondrosis is a cartilaginous union between two non-mobile bones that allows the area to grow until the cartilage is mineralized. It is an important anatomical location of cranial growth that also influences the development of the mandible and the maxilla [7, 8]. Cranial base growth after birth continues until adolescence, especially in the area of the spheno-occipital synchondrosis. The beginning of the closure of this synchondrosis is related to the onset of puberty in teenagers [9].

On conventional skull radiographs, it is not possible to determine when this synchondrosis begins to close or whether it is closed totally because of the superimposition of adjacent structures, which is one of the inherent limitations of two-dimensional imaging. The closure of this synchondrosis has been typically studied through direct inspection on cadavers [10, 11]; and more recently, CBCT has been used to analyse the closure of this synchondrosis [12–14].

The maturational stage of a subject can be estimated by analysing the closure of the spheno-occipital synchondrosis according to a previously defined scoring method of synchondrosis fusion. Fusion status has been scored according to a four-[14] or five-stage method [12] in the midsagittal plane, or a six-stage method [13] when both the sagittal and axial planes are evaluated.

Considering the clinical applicability of skeletal maturation status provided by the CVM method [4, 15–19], it seems logical to investigate the correlation between the closure time of the spheno-occipital synchondrosis and CVM stage in growing and young adult subjects. If a correlation is confirmed, the closure status of spheno-occipital synchondrosis might be an additional resource that could be used to determine the skeletal maturation status of a given patient accurately.

The development of contemporary imaging technologies such as cone beam computed tomography (CBCT) allows us to study important osseous structures in detail and in all three planes of space, complementing our knowledge about these structures while dramatically reducing the radiation dosage when compared to other advanced imaging modalities such multi-slice medical computed tomography [20]. These three-dimensional imaging modalities are important tools that allow the non-destructive analysis of otherwise non-accessible anatomical structures such as the spheno-occipital synchondrosis.

Thus the aim of this study was to determine the relationship between the closure stage of spheno-occipital synchondrosis and CVM stage in growing and young adult subjects using CBCT.

## Materials and Methods

### Study population

The sample size was determined by using G*Power v3.3 (http://www.gpower.hhu.de/en.html), assuming a correlation between vertebrae and synchondrosis stages of 0.20, power of 0.95, and type I error of 0.05. According to this power analysis, the number of patients required was 266. In order to achieve this number, a multicenter study was designed. The study was approved by the appropriate Institutional Review Board of the University of Michigan (HUM00062392), that issued an exemption to this study because of the use of collected existing data in such a manner that subjects cannot be identified, directly or through identifiers linked to the subjects. All of the patients or their legal tutors signed an informed consent for their respective treatments.

A total of 315 participants (148 females and 167 males; mean age = 15.6 years; median = 13.7; SD = 7.3) were selected from two different centers, The University of Michigan (n = 115) and a private orthodontic practice in Miami, FL (n = 200). Inclusion criteria were: Age between 7 and 25 years, white, and an extended field of view (FOV) CBCT. The exclusion criteria were syndromes affecting the craniofacial and cervical column structures or history of trauma to the head and neck, and previous history of head and neck surgery.

### Image acquisition

The CBCT scans that were included in this study were selected from a database of scans that had been previously acquired for orthodontic diagnosis and treatment planning purposes. All scans were acquired using an i-CAT CBCT machine (Imaging Sciences International, Hatfield, PA, USA). The imaging parameters were set at 120 kVp, 18.66 mAs, scan time 20 seconds, resolution 0.4 mm, and 13 mm x 10 mm field of view. The DICOM files of each CBCT scan were exported and transferred to a desktop computer equipped with a DICOM viewer software called InVivo 5 (Anatomage, San Jose, CA, USA).

The CBCT-generated lateral CVM images were obtained using this software under the same gray-scale condition. Any head tilt of the patient in the CBCT image was corrected on the computer so that the midsagittal plane perpendicular to the floor was running through the intermaxillary suture and anterior nasal spine.

### Classification methods

**Fig 1:**
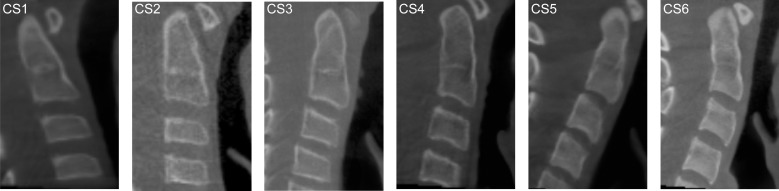
Cervical vertebrae maturational stages (CVM method). A) CS1 B) CS2 C) CS3 D) CS4 E) CS5 F) CS6).

CVM stages were defined according to the method by Baccetti et al. [4]. Taking into account only the C2 to C4 vertebral bodies, this method defines 6 different stages of cervical vertebral maturation based on shape modifications of these vertebrae. The cervical stages (CS) are as follow (Fig 1): In CS1, the lower borders of all the three vertebrae (C2-C4) are flat, and the bodies of both C3 and C4 are trapezoid in shape (the superior border of the vertebral body is tapered from posterior to anterior). In CS2, a concavity is present at the lower border of C2 and the bodies of both C3 and C4 are still trapezoidal in shape. In CS3, concavities at the lower borders of both C2 and C3 are present, and the bodies of C3 and C4 may be either trapezoidal or rectangular horizontal in shape. In CS4, concavities at the lower border of C2, C3, and C4 are now present and the bodies of both C3 and C4 are rectangular horizontal in shape. In CS5, the concavities at the lower border of C2, C3, and C4 are still present and at least one of the bodies of C3 and C4 is squared in shape; if not squared, the body of the other cervical vertebra is still rectangular horizontal. In CS6, the concavities at the lower borders of C2, C3, and C4 still are evident and at least one of the bodies of C3 and C4 is rectangular vertical in shape; if not rectangular vertical, the body of the other cervical vertebra is squared. CS1 and CS2 have been reported to be reached typically before the pubertal growth spurt, CS3 and CS4 have been reported to occur in coincidence with the pubertal growth spurt, CS3 with its onset, and CS4 after the peak height velocity, with CS5 and CS6 occurring after the growth spurt [4].

**Fig 2:**
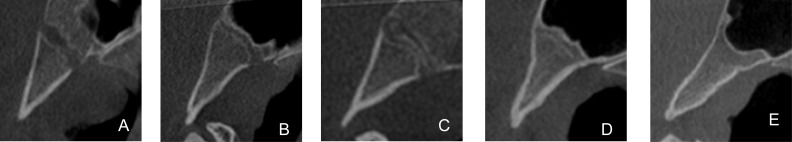
Spheno-occipital synchondrosis fusion stages. A) Stage 1. B) Stage 2. C) Stage 3. D) Stage 4. E) Stage 5.

The ossification status of the spheno-occipital synchondrosis was assessed using the recent five-stage system developed by Bassed et al. [12]. The stages are as follows (Fig 2): In stage 1, the synchondrosis is completely open and unfused. In stage 2, the superior border has fused, while the remainder of the fusion site is patent. In stage 3, half the length of the synchondrosis is closed. In stage 4, closure is essentially complete, but the site is still visible by way of a fusion scar. In stage 5, the site has been completely obliterated with the appearance of normal bone throughout.

### Statistical analysis

The relationship between synchondrosis and cervical vertebrae stages was analyzed using Spearman’s non-parametric correlation to avoid non-normality, as age does not follow normality (Shapiro-Wilk p-value = 0.008). Ordinal regression was used to study the ability of the synchondrosis stage to predict the vertebral stage, including age and sex as additional factors, and using a logit link function. The logit was chosen as the link function because differences between adjacent stages cannot be considered equivalent along the whole classification system [21]. For example, the distance between stage 1 and 2 cannot be assumed equal to the distance between stages 2 and 3.

All images were scored by a single, experienced observer (MJF). To test for observer reliability, 50 randomly selected images were scored by another independent expert (JAA). Inter- and intra-rater agreements were calculated using Cohen’s Kappa coefficient.

All statistical analyses were performed using IBM SPSS v20.0 (IBM).

## Results

The inter and intra-rater agreement coefficients were almost perfect for both, vertebrae (k = 0.90, and k = 0.95 respectively) and suture (k = 0.92, and k = 0.92 respectively) [22].

Table 1 displays the frequencies of stages of vertebrae and synchondrosis as well as the cross-tabulation of vertebrae by synchondrosis. Individual scores of cervical vertebrae and spheno-occipital synchondrosis maturational stages are given in S1 Table.

**Table 1:** Frequencies of stages of vertebra and synchondrosis, and vertebrae by synchondrosis.

	Synchondrosis Stages					
Vertebra Stages	1	2	3	4	5	Total Vertebra
1	31	1	0	0	0	32
2	39	10	0	0	0	49
3	27	26	11	0	0	64
4	0	12	33	14	0	59
5	0	1	19	60	1	81
6	0	0	0	27	3	30
Total Synchondrosis	97	50	63	101	4	315

Age was significantly correlated with vertebrae (r = 0.66, p < .001) and synchondrosis (r = 0.66 p < .001). Sex also showed a low but significant association with synchondrosis (r = 0.15, p = .008) and vertebrae (r = .12, p = .05). Vertebrae and synchondrosis have a strong significant correlation (r = 0.89, p < .001) that was similar for females (r = 0.88, p < .001) and males (r = 0.89, p < .001), as was the correlation of age with synchondrosis (r = 0.73, and r = 0.65, for females and males respectively, p < .001), and with vertebra (r = 0.70, and r = 0.65, for females and males respectively, p < .001).

Given that sex seemed to not affect the association of vertebra and synchondrosis, the next ordinal regression analysis [23] was performed for the whole sample. Ordinal regression indicated that the cervical vertebral maturation stage can be accurately predicted from the synchondrosis stage, χ²(6) = 457.54, p < .001 (Cox-Snell pseudo R² = 0.77). No differences between slope parameters were observed, χ²(24) = 27.35, p = 0.29. This model is able to estimate the probability of being at a vertebral maturation stage Yi = k (k = 1…6), after knowing the synchondrosis stage, sex, and biological age as follows:

$$Z_{i}=\sum_{i=1}^{k}X_{i}L,$$

with X_i_ being the vector containing synchondrosis stage, age and sex of participant i, and L the locations vector (Table 2). Individual probabilities of being in a given vertebra stage (Y_i_) then are computed as follows:

$$P(Y_{i}=k)=\frac{1}{1+e^{z_{i}-T_{k}}}-\sum_{j=1}^{k-1}\frac{1}{1+e^{z_{i-T_{j}}}}$$

with T_j_ being the classification threshold for vertebral stage j (Table 2).

**Table 2:** Estimates of the multivariate ordinal regression model for the prediction of vertebra stage from the synchondrosis stage, plus sex (categorical) and age (continuous) variables.

	Vertebra	Estimate	SE	LL	UL	Wald χ²	P
Threshold	1	-9.56	1.43	-12.36	-6.76	44.77	.00
	2	-7.72	1.41	-10.48	-4.95	29.82	.00
	3	-4.83	1.37	-7.51	-2.15	12.45	.00
	4	-2.19	1.33	-4.79	0.42	2.70	.10
	5	1.23	1.32	-1.36	3.82	0.87	.35
Location	Age (years)	0.10	0.02	0.06	0.15	20.50	.00
	Sex	-0.15	0.24	-0.61	0.32	0.39	.53
	Synchondrosis						
	1	-9.98	1.37	-12.67	-7.30	53.12	.00
	2	-7.35	1.34	-9.97	-4.72	30.00	.00
	3	-4.47	1.29	-6.99	-1.95	12.06	.00
	4	-2.01	1.24	-4.44	.42	2.62	.11

Note: LL: lower limit of the 95% confidence interval, UL: upper bound of the 95% confidence interval.

The application of the above probability equations is straightforward. For example, consider a 14 years old male showing a synchondrosis stage of 4. The probability of this person to be in vertebra stage 5 is 0.86, given that:

$$Z_{i}=\sum_{i=1}^{k}X_{i}L=x_{1}L_{1}+x_{2}L_{2}{+x}_{3}L_{3}=(1)(-2.01)+(14)(0.103)+(1)(-0.148)=1.62$$

and

$$P(Y_{i}=5)=\frac{1}{1+e^{z_{i}-T_{k}}}=\frac{1}{1+e^{\left(1.62-1.232)\right)}}=0.86$$

Thus this child is predicted to be in cervical stage 5, as the probabilities of being in the remaining stages are clearly smaller than that (note that the sum of probabilities sum to unity).

We next predicted the vertebral stage category for each of the study participants, and then we tested the goodness-of-fit of the prediction by χ², using a Montecarlo approach (10,000 random samples) to derive statistical significance. Results indicate that the prediction fitted significantly the observed vertebral stages, χ² = 440.10, p<0.001(Table 3).

**Table 3:** Percentages of observed versus predicted vertebra stage.

	Predicted					
Observed	1	2	3	4	5	6
1	12.5	84.4	3.1	0.0	0.0	0.0
2	0.0	79.6	20.4	0.0	0.0	0.0
3	0.0	42.2	40.6	17.2	0.0	0.0
4	0.0	0.0	20.3	54.2	25.4	0.0
5	0.0	0.0	1.2	23.5	66.7	8.6
6	0.0	0.0	0.0	0.0	56.7	43.3

Finally, we performed a cross-validation analysis to test for model generality by dividing the whole sample randomly in two halves, estimating the model parameters in one half and applying the estimated parameters to the other half of the sample. Using this approach, we observed highly significant classifications of vertebral from synchondrosis stages plus age, χ²(20) = 371.86, p<0.001, indicating that the prediction matched significantly the observed vertebral stages, χ² = 440.10, p<0.001.

## Discussion

The present study demonstrated diagnostic agreement between the different stages of maturation of the spheno-occipital synchondrosis and those of the cervical vertebrae in growing and young adult white subjects. Our results demonstrate that both variables are correlated significantly, enabling us to establish a prediction model between them according to the gender and age of the patients.

The method reported in our study is useful in many fields of Dentistry (i.e. Orthodontics, Implant Dentistry, Maxillofacial Surgery, etc.), but it could also have interesting applicability in other areas of the Science, for example in Pediatrics, Forensic Medicine, Anthropology or Paleo-Anthropology; i.e. when only human remains including spheno-occipital anatomic area are disposable.

Although there was some controversy about the use of CBCT scans to assess CVM stages [24], nevertheless recent studies showed a good relationship between skeletal maturation assessed by cervical vertebrae maturational method as seen on CBCT, and lateral cephalogram [25], and later contemporary studies also support these results and estimated skeletal maturation from cervical vertebrae images obtained from CBCT scans [26–28]

The CVM method to determine the growth stage of a given subject has shown to have some concerns, which could make it difficult to be applied in the daily clinical practice. Some studies have reported that the CVM method has a low to good intra-rater reproducibility [15, 16, 29–31], nevertheless, when specific training is provided along with precise guidelines in assessing visually each stage, the CVM method proves to be accurate and repeatable to a satisfactory level [31].

On the other hand, the spheno-occipital synchondrosis is easy to locate, and its stage of maturation can be determined by means of CBCT imaging. Thus, the method reported in the present study, which is based on the closure status of the spheno-occipital synchondrosis, could be a new method to determine the skeletal maturation status of a given patient, when a CBCT is available, e.g. as a pre-treatment record. This approach would also avoid the need of a hand-wrist radiograph to determine the growth stage of the subject.

The spheno-occipital synchondrosis has gained importance for both age estimation and clinical applications, particularly because of its late ossification process [32]. Fusion of the spheno-occipital synchondrosis usually starts about 2 years earlier in females than in males [11, 33], in a similar way than that observed during general growth at the beginning of puberty [34]; however, no significant differences seem to occur at the end of the process [12, 32].

Age estimation of subjects by using the closure stage of spheno-occipital synchondrosis is widely variable among studies, which may be attributed to the different methodological approaches used, i.e. macroscopic [11, 35, 36], radiographic [37] or CBCT [12–14, 32], different scoring systems, sex, or population variations.

Although of great interest in forensic and anthropologic sciences, age estimation of subjects with spheno-occipital synchondrosis was not the aim of our study. Rather, the current study focused on skeletal maturity stage estimation, and thus, was not affected by this disparity in reported closure age and complete ossification age [10–14, 32, 33, 37–39].

Spheno-occipital synchondrosis fusion status has been scored according to the five-stage system by Basset et al. [12], which considered the mid-sagittal plane of the skull base as the view of choice. In this novel method, stage 4 is a new growth stage added specifically due to the ability to visualize the fusion scar on high resolution CBCT images, which is not possible with conventional radiography or in dried skulls. This staging system was chosen as the different stages are easily definable when viewed on CBCT and reproducible in terms of both intra- and inter-rater examination [12]. In addition, CBCT records employed in the present study offer high resolution images that allow a detailed examination of spheno-occipital synchondrosis staging, while reducing the radiation dosage when compared to conventional computed tomography [20].

In the present study, the maturation of the cervical vertebrae and the spheno-occipital synchondrosis were shown to have a strong association. The non-parametric Spearman's rho of 0.89 indicated very good overall agreement between the closure stage of the spheno-occipital synchondrosis and the CVM stage. Based on these results, the synchondrosis stage can accurately predict the vertebral stage in a given individual.

Additionally, our study focused on developing an ordinal prediction equation of the vertebral stage using the synchondrosis stage, sex, and biological age as predictors. Our cross-validation approach suggests that the predictive ability of our ordinal regression model is robust and can be generalized to other populations, at least within the age ranges included in this study. In addition, spheno-occipital synchondrosis closure stage could be used to estimate the growth stage in individual subjects, which may have important clinical applications.

From a clinical perspective, it is of great interest, for example, that pubertal growth spurt has been reported to coincide with CS4, which correspond to spheno-occipital closure stage 3. Therefore, the spheno-occipital synchondrosis closure stage could be useful for planning treatment timing for skeletal Class II or III malocclusion and maxillary constriction [4, 40]. Similarly, the beginning of closure of the spheno-occipital synchondrosis (stage 1) coincides with the onset of the pubertal growth spurt.

As it is supposed, we do not recommend to take a complete cranial CBCT scan from every patient. Instead of, we claim the utility of previous CBCTs of a given patient when they are disposable for other clinical reasons. For example, in cases of impacted canines, or problems generated by wisdom teeth (very common situations in the daily clinical practice), it is recommended a reduced CBCT scan, that usually include the spheno-occipital synchondrosis area. In these cases, the cervical vertebrae are not included in the CBCTs, but the correlation with CVM stages, and consequently skeletal maturation, could be obtained with our proposed method, from spheno-occipital synchondrosis closure stage. Obviously, the radiation dose of our patients was also reduced, if we avoid to use additional radiographic techniques to obtain this important information.

Thus, when a CBCT is available for diagnostic purposes, or when the lateral cephalogram is of insufficient quality to determine the stage of cervical vertebrae development, i.e. the cervical vertebrae might be partially covered by the thyroid collar or lead apron used to reduce the radiation exposure, then growth stage maturation in growing and young adult subjects could be estimated in a straightforward manner by evaluating the spheno-occipital synchondrosis stage and by using the equations developed in the current study.

## Conclusions

The time of closure of spheno-occipital synchondrosis and cervical vertebrae maturation stage are highly correlated using CBCT techniques. The spheno-occipital synchondrosis stage appears to be a good indicator of growth maturation.

## Supporting Information

S1 Table Individual scores of cervical vertebrae and spheno-occipital synchondrosis maturational stages. (XLSX)
